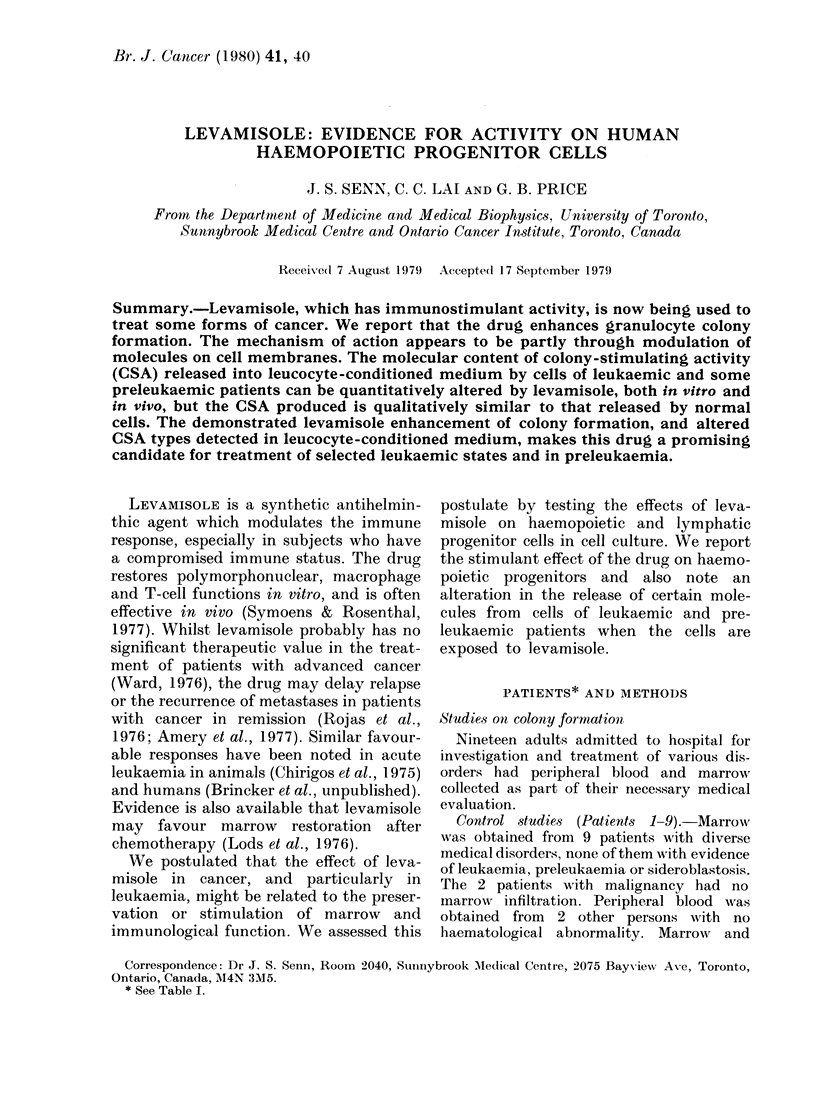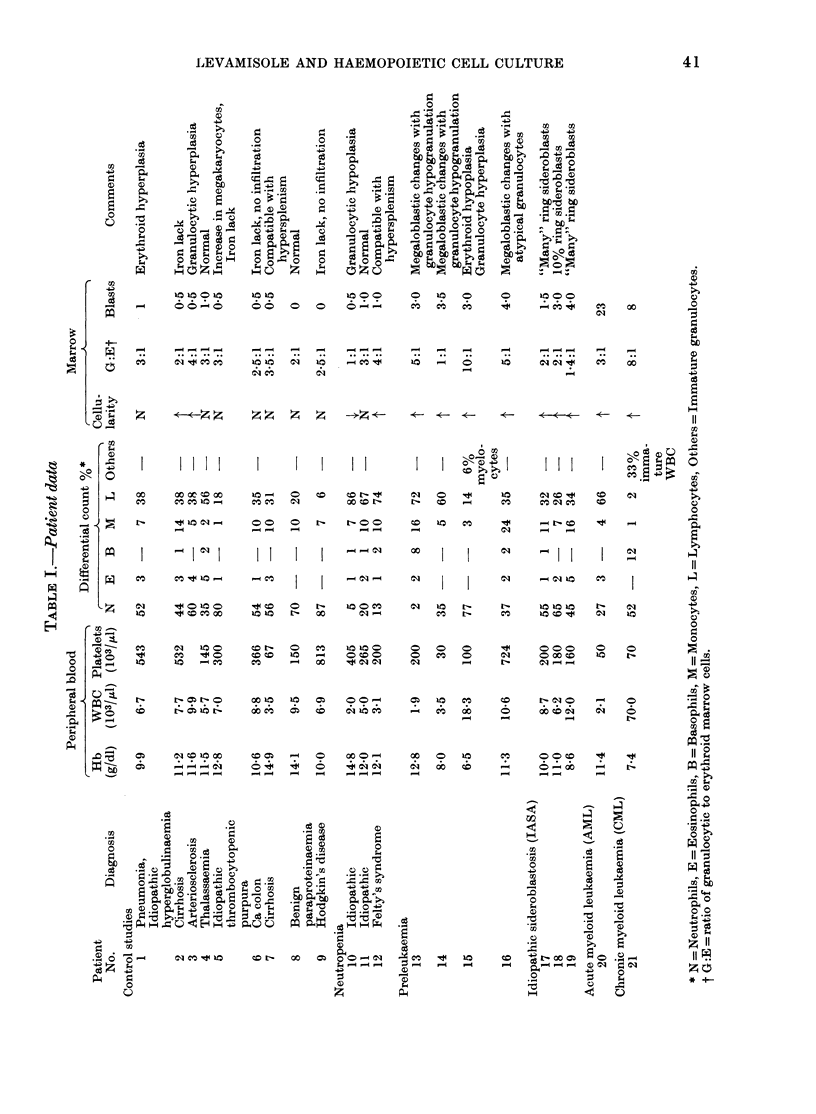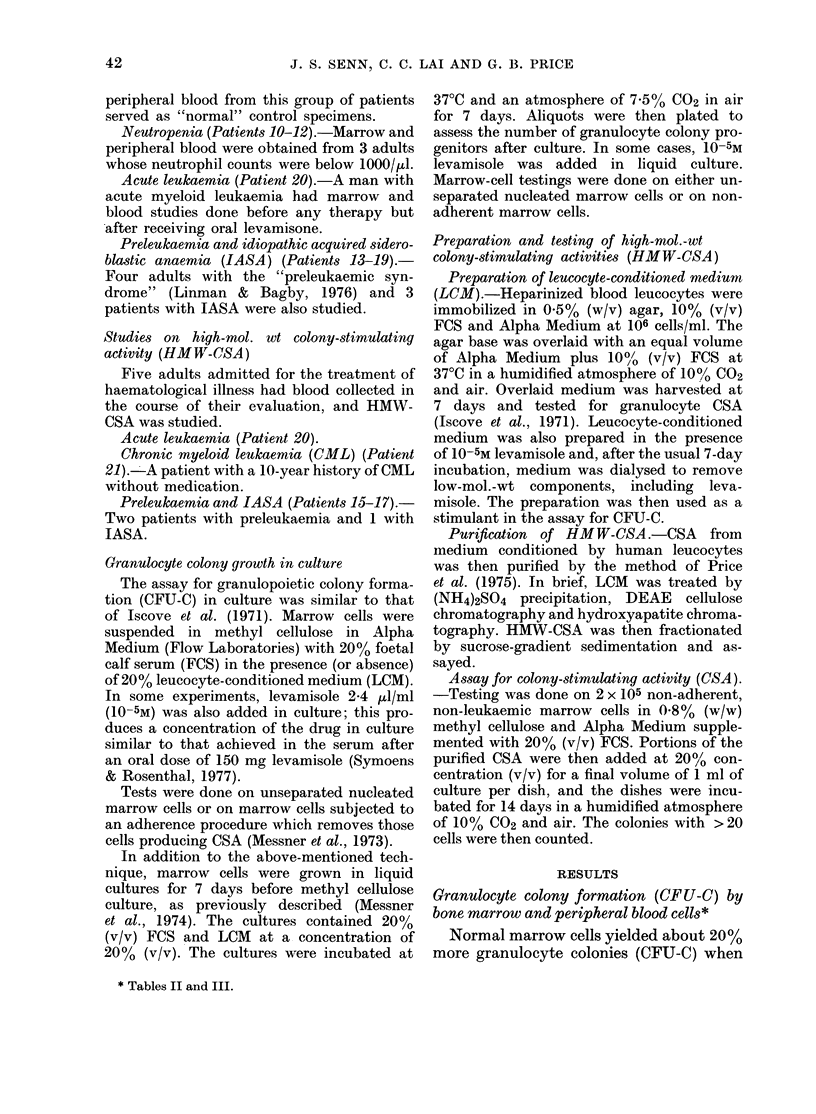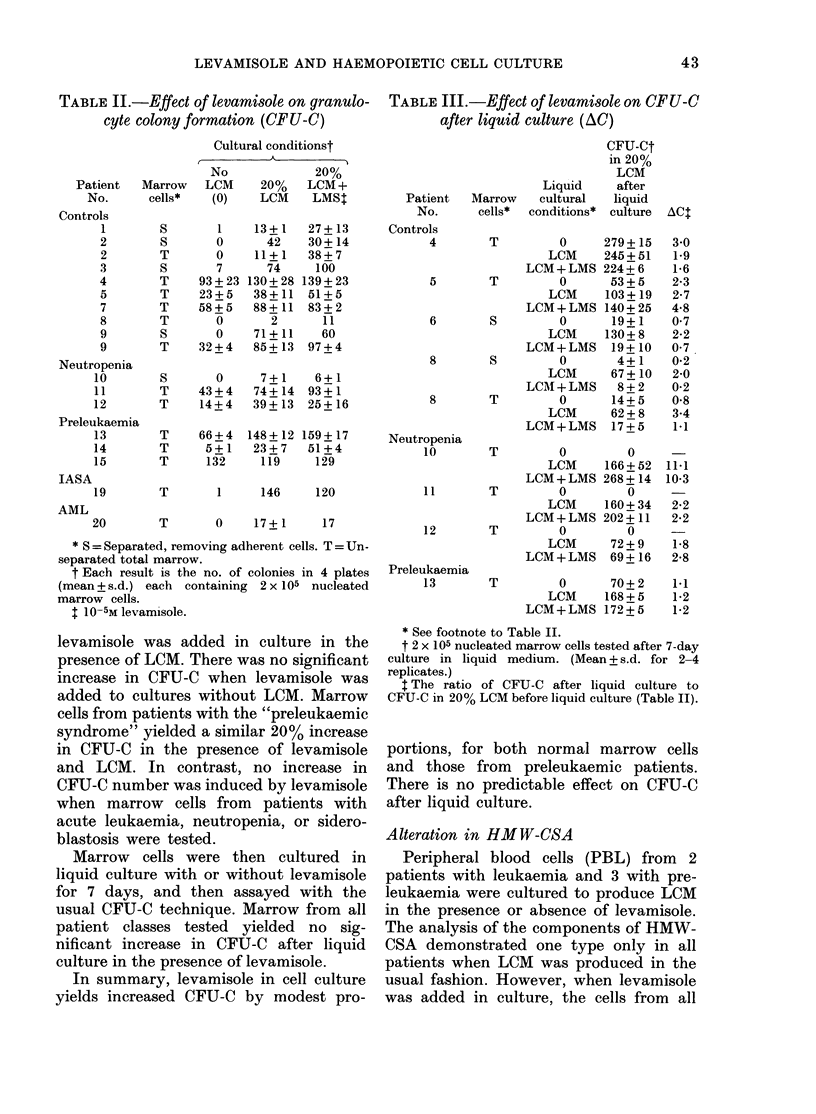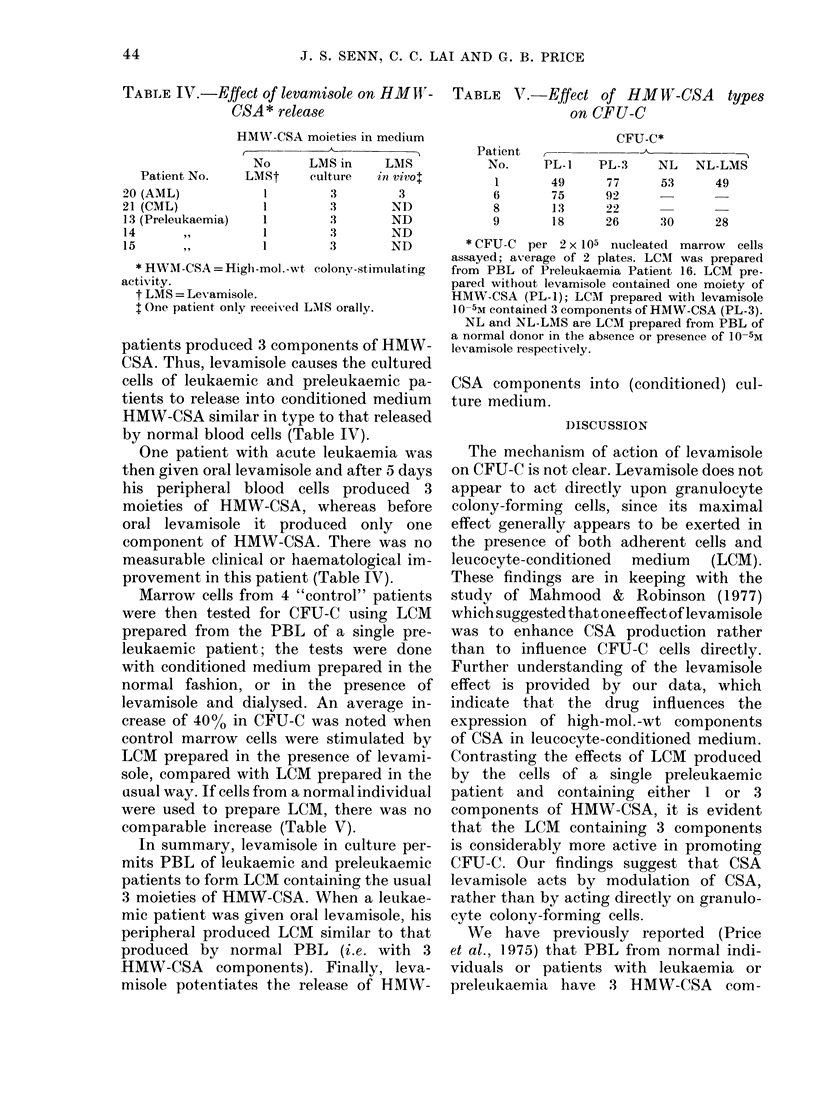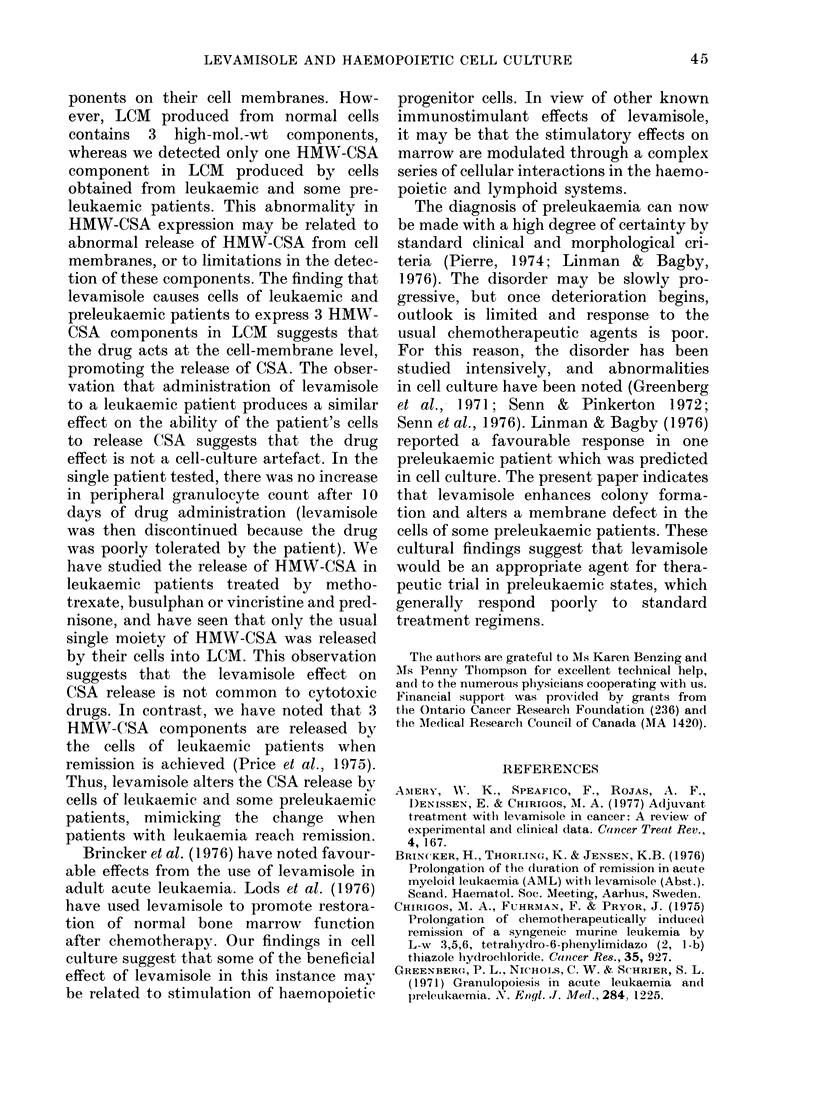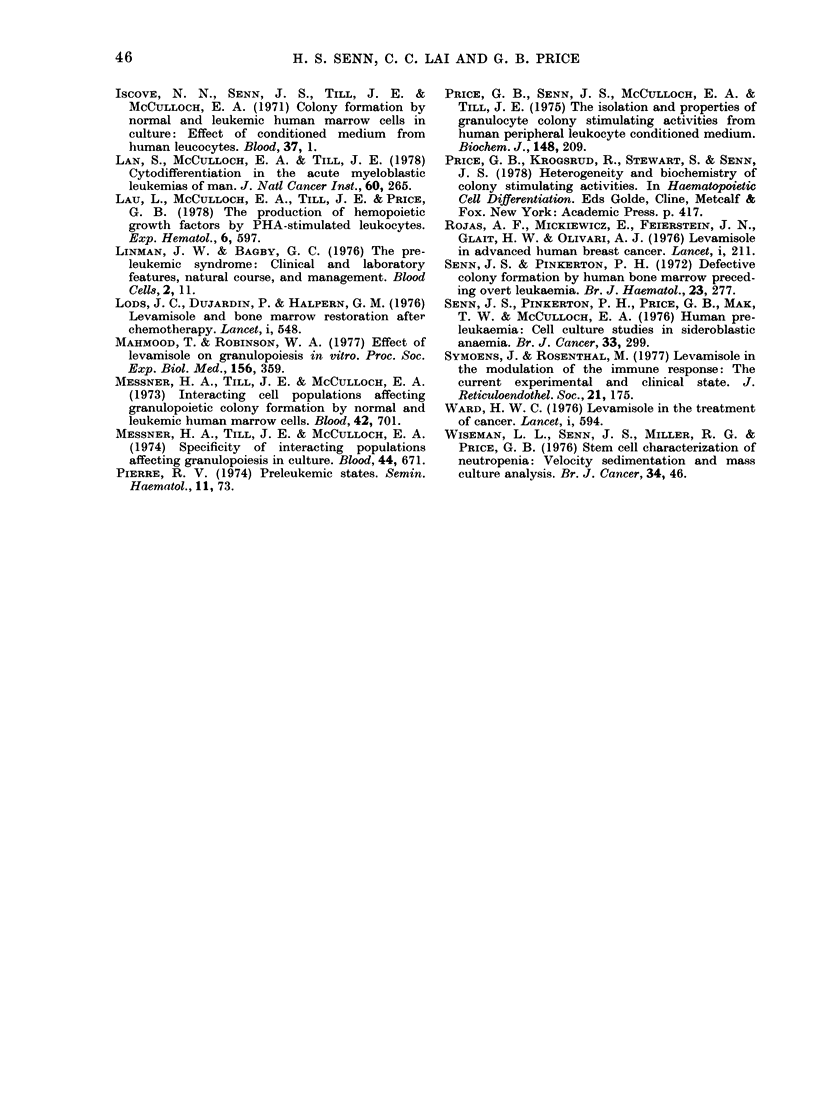# Levamisole: evidence for activity on human haemopoietic progenitor cells.

**DOI:** 10.1038/bjc.1980.5

**Published:** 1980-01

**Authors:** J. S. Senn, C. C. Lai, G. B. Price

## Abstract

Levamisole, which has immunostimulant activity, is now being used to treat some forms of cancer. We report that the drug enhances granulocyte colony formation. The mechanism of action appears to be partly through modulation of molecules on cell membranes. The molecular content of colony-stimulating activity (CSA) released into leucocyte-conditioned medium by cells of leukaemic and some preleukaemic patients can be quantitatively altered by levamisole, both in vitro and in vivo, but the CSA produced is qualitatively similar to that released by normal cells. The demonstrated levamisole enhancement of colony formation, and altered CSA types detected in leucocyte-conditioned medium, makes this drug a promising candidate for treatment of selected leukaemic states and in preleukaemia.


*Br. J. Cancer* (1980) **41**, 40

### LEVAMISOLE: EVIDENCE FOR ACTIVITY ON HUMAN HAEMOPOIETIC PROGENITOR CELLS

J. S. SENN, C. C. LAI AND G. B. PRICE

*From the Department of Medicine and Medical Biophysics, University of Toronto, Sunnybrook Medical Centre and Ontario Cancer Institute, Toronto, Canada*

Received 7 August 1979 Accepted 17 September 1979

**Summary.—Levamisole, which has immunostimulant activity, is now being used to treat some forms of cancer. We report that the drug enhances granulocyte colony formation. The mechanism of action appears to be partly through modulation of molecules on cell membranes. The molecular content of colony-stimulating activity (CSA) released into leucocyte-conditioned medium by cells of leukaemic and some preleukaemic patients can be quantitatively altered by levamisole, both *in vitro* and *in vivo*, but the CSA produced is qualitatively similar to that released by normal cells. The demonstrated levamisole enhancement of colony formation, and altered CSA types detected in leucocyte-conditioned medium, makes this drug a promising candidate for treatment of selected leukaemic states and in preleukaemia.**

LEVAMISOLE is a synthetic antihelminthic agent which modulates the immune response, especially in subjects who have a compromised immune status. The drug restores polymorphonuclear, macrophage and T-cell functions *in vitro*, and is often effective *in vivo* (Symoens & Rosenthal, 1977). Whilst levamisole probably has no significant therapeutic value in the treatment of patients with advanced cancer (Ward, 1976), the drug may delay relapse or the recurrence of metastases in patients with cancer in remission (Rojas *et al.*, 1976; Amery *et al.*, 1977). Similar favourable responses have been noted in acute leukaemia in animals (Chirigos *et al.*, 1975) and humans (Brincker *et al.*, unpublished). Evidence is also available that levamisole may favour marrow restoration after chemotherapy (Lods *et al.*, 1976).

We postulated that the effect of levamisole in cancer, and particularly in leukaemia, might be related to the preservation or stimulation of marrow and immunological function. We assessed this postulate by testing the effects of levamisole on haemopoietic and lymphatic progenitor cells in cell culture. We report the stimulant effect of the drug on haemopoietic progenitors and also note an alteration in the release of certain molecules from cells of leukaemic and preleukaemic patients when the cells are exposed to levamisole.

### PATIENTS* AND METHODS

### *Studies on colony formation*

Nineteen adults admitted to hospital for investigation and treatment of various disorders had peripheral blood and marrow collected as part of their necessary medical evaluation.

*Control studies* (*Patients 1–9*).—Marrow was obtained from 9 patients with diverse medical disorders, none of them with evidence of leukaemia, preleukaemia or sideroblastosis. The 2 patients with malignancy had no marrow infiltration. Peripheral blood was obtained from 2 other persons with no haematological abnormality. Marrow and

Correspondence: Dr J. S. Senn, Room 2040, Sunnybrook Medical Centre, 2075 Bayview Ave, Toronto, Ontario, Canada, M4N 3M5.

* See Table I.

TABLE I.—*Patient data*

| Patient No. | Diagnosis | Peripheral blood: Hb (g/dl) | WBC ($10^3/\mu l$) | Platelets ($10^3/\mu l$) | Differential count %*: N | E | B | M | L | Others | Marrow: Cellularity | G:E† | Blasts | Comments |
|---|---|---|---|---|---|---|---|---|---|---|---|---|---|---|
| Control studies | | | | | | | | | | | | | | |
| 1 | Pneumonia, Idiopathic hyperglobulinaemia | 9·9 | 6·7 | 543 | 52 | 3 | — | 7 | 38 | — | N | 3:1 | 1 | Erythroid hyperplasia |
| 2 | Cirrhosis | 11·2 | 7·7 | 532 | 44 | 3 | 1 | 14 | 38 | — | ↑ | 2:1 | 0·5 | Iron lack |
| 3 | Arteriosclerosis | 11·6 | 9·9 | | 60 | 4 | — | 5 | 38 | — | ↑ | 4:1 | 0·5 | Granulocytic hyperplasia |
| 4 | Thalassaemia | 11·5 | 5·7 | 145 | 35 | 5 | 2 | 2 | 56 | — | N | 3:1 | 1·0 | Normal |
| 5 | Idiopathic thrombocytopenic purpura | 12·8 | 7·0 | 300 | 80 | 1 | — | 1 | 18 | — | N | 3:1 | 0·5 | Increase in megakaryocytes, Iron lack |
| 6 | Ca colon | 10·6 | 8·8 | 366 | 54 | 1 | — | 10 | 35 | — | N | 2·5:1 | 0·5 | Iron lack, no infiltration |
| 7 | Cirrhosis | 14·9 | 3·5 | 67 | 56 | 3 | — | 10 | 31 | | N | 3·5:1 | 0·5 | Compatible with hypersplenism |
| 8 | Benign paraproteinaemia | 14·1 | 9·5 | 150 | 70 | — | — | 10 | 20 | — | N | 2:1 | 0 | Normal |
| 9 | Hodgkin's disease | 10·0 | 6·9 | 813 | 87 | — | — | 7 | 6 | — | N | 2·5:1 | 0 | Iron lack, no infiltration |
| Neutropenia | | | | | | | | | | | | | | |
| 10 | Idiopathic | 14·8 | 2·0 | 405 | 5 | 1 | 1 | 7 | 86 | — | ↓ | 1:1 | 0·5 | Granulocytic hypoplasia |
| 11 | Idiopathic | 12·0 | 5·0 | 265 | 20 | 2 | 1 | 10 | 67 | — | N | 3:1 | 1·0 | Normal |
| 12 | Felty's syndrome | 12·1 | 3·1 | 200 | 13 | 1 | 2 | 10 | 74 | | ↑ | 4:1 | 1·0 | Compatible with hypersplenism |
| Preleukaemia | | | | | | | | | | | | | | |
| 13 | | 12·8 | 1·9 | 200 | 2 | 2 | 8 | 16 | 72 | — | ↑ | 5:1 | 3·0 | Megaloblastic changes with granulocyte hypogranulation |
| 14 | | 8·0 | 3·5 | 30 | 35 | — | — | 5 | 60 | — | ↑ | 1:1 | 3·5 | Megaloblastic changes with granulocyte hypogranulation |
| 15 | | 6·5 | 18·3 | 100 | 77 | — | — | 3 | 14 | 6% myelocytes | ↑ | 10:1 | 3·0 | Erythroid hypoplasia Granulocyte hyperplasia |
| 16 | | 11·3 | 10·6 | 724 | 37 | 2 | 2 | 24 | 35 | — | ↑ | 5:1 | 4·0 | Megaloblastic changes with atypical granulocytes |
| Idiopathic sideroblastosis (IASA) | | | | | | | | | | | | | | |
| 17 | | 10·0 | 8·7 | 200 | 55 | 1 | 1 | 11 | 32 | — | ↑ | 2:1 | 1·5 | "Many" ring sideroblasts |
| 18 | | 11·0 | 6·2 | 180 | 65 | 2 | — | 7 | 26 | — | ↑ | 2:1 | 3·0 | 10% ring sideroblasts |
| 19 | | 8·6 | 12·0 | 160 | 45 | 5 | — | 16 | 34 | — | ↑ | 1·4:1 | 4·0 | "Many" ring sideroblasts |
| Acute myeloid leukaemia (AML) | | | | | | | | | | | | | | |
| 20 | | 11·4 | 2·1 | 50 | 27 | 3 | — | 4 | 66 | — | ↑ | 3:1 | 23 | |
| Chronic myeloid leukaemia (CML) | | | | | | | | | | | | | | |
| 21 | | 7·4 | 70·0 | 70 | 52 | — | 12 | 1 | 2 | 33% immature WBC | ↑ | 8:1 | 8 | |

* N = Neutrophils, E = Eosinophils, B = Basophils, M = Monocytes, L = Lymphocytes, Others = Immature granulocytes.

† G:E = ratio of granulocytic to erythroid marrow cells.

peripheral blood from this group of patients served as "normal" control specimens.

*Neutropenia* (*Patients 10–12*).—Marrow and peripheral blood were obtained from 3 adults whose neutrophil counts were below 1000/$\mu$l.

*Acute leukaemia* (*Patient 20*).—A man with acute myeloid leukaemia had marrow and blood studies done before any therapy but after receiving oral levamisone.

*Preleukaemia and idiopathic acquired sideroblastic anaemia* (*IASA*) (*Patients 13–19*).—Four adults with the "preleukaemic syndrome" (Linman & Bagby, 1976) and 3 patients with IASA were also studied.

*Studies on high-mol. wt colony-stimulating activity* (*HMW-CSA*)

Five adults admitted for the treatment of haematological illness had blood collected in the course of their evaluation, and HMW-CSA was studied.

*Acute leukaemia* (*Patient 20*).

*Chronic myeloid leukaemia* (*CML*) (*Patient 21*).—A patient with a 10-year history of CML without medication.

*Preleukaemia and IASA* (*Patients 15–17*).—Two patients with preleukaemia and 1 with IASA.

*Granulocyte colony growth in culture*

The assay for granulopoietic colony formation (CFU-C) in culture was similar to that of Iscove *et al.* (1971). Marrow cells were suspended in methyl cellulose in Alpha Medium (Flow Laboratories) with 20% foetal calf serum (FCS) in the presence (or absence) of 20% leucocyte-conditioned medium (LCM). In some experiments, levamisole 2·4 $\mu$l/ml ($10^{-5}$M) was also added in culture; this produces a concentration of the drug in culture similar to that achieved in the serum after an oral dose of 150 mg levamisole (Symoens & Rosenthal, 1977).

Tests were done on unseparated nucleated marrow cells or on marrow cells subjected to an adherence procedure which removes those cells producing CSA (Messner *et al.*, 1973).

In addition to the above-mentioned technique, marrow cells were grown in liquid cultures for 7 days before methyl cellulose culture, as previously described (Messner *et al.*, 1974). The cultures contained 20% (v/v) FCS and LCM at a concentration of 20% (v/v). The cultures were incubated at 37°C and an atmosphere of 7·5% $CO_2$ in air for 7 days. Aliquots were then plated to assess the number of granulocyte colony progenitors after culture. In some cases, $10^{-5}$M levamisole was added in liquid culture. Marrow-cell testings were done on either unseparated nucleated marrow cells or on non-adherent marrow cells.

*Preparation and testing of high-mol.-wt colony-stimulating activities* (*HMW-CSA*)

*Preparation of leucocyte-conditioned medium* (*LCM*).—Heparinized blood leucocytes were immobilized in 0·5% (w/v) agar, 10% (v/v) FCS and Alpha Medium at $10^6$ cells/ml. The agar base was overlaid with an equal volume of Alpha Medium plus 10% (v/v) FCS at 37°C in a humidified atmosphere of 10% $CO_2$ and air. Overlaid medium was harvested at 7 days and tested for granulocyte CSA (Iscove *et al.*, 1971). Leucocyte-conditioned medium was also prepared in the presence of $10^{-5}$M levamisole and, after the usual 7-day incubation, medium was dialysed to remove low-mol.-wt components, including levamisole. The preparation was then used as a stimulant in the assay for CFU-C.

*Purification of HMW-CSA*.—CSA from medium conditioned by human leucocytes was then purified by the method of Price *et al.* (1975). In brief, LCM was treated by $(NH_4)_2SO_4$ precipitation, DEAE cellulose chromatography and hydroxyapatite chromatography. HMW-CSA was then fractionated by sucrose-gradient sedimentation and assayed.

*Assay for colony-stimulating activity* (*CSA*).—Testing was done on $2 \times 10^5$ non-adherent, non-leukaemic marrow cells in 0·8% (w/w) methyl cellulose and Alpha Medium supplemented with 20% (v/v) FCS. Portions of the purified CSA were then added at 20% concentration (v/v) for a final volume of 1 ml of culture per dish, and the dishes were incubated for 14 days in a humidified atmosphere of 10% $CO_2$ and air. The colonies with $>20$ cells were then counted.

### RESULTS

*Granulocyte colony formation* (*CFU-C*) *by bone marrow and peripheral blood cells**

Normal marrow cells yielded about 20% more granulocyte colonies (CFU-C) when

* Tables II and III.

TABLE II.—*Effect of levamisole on granulocyte colony formation (CFU-C)*

| Patient No. | Marrow cells* | Cultural conditions† No LCM (0) | 20% LCM | 20% LCM+LMS‡ |
|---|---|---|---|---|
| Controls | | | | |
| 1 | S | 1 | 13±1 | 27±13 |
| 2 | S | 0 | 42 | 30±14 |
| 2 | T | 0 | 11±1 | 38±7 |
| 3 | S | 7 | 74 | 100 |
| 4 | T | 93±23 | 130±28 | 139±23 |
| 5 | T | 23±5 | 38±11 | 51±5 |
| 7 | T | 58±5 | 88±11 | 83±2 |
| 8 | T | 0 | 2 | 11 |
| 9 | S | 0 | 71±11 | 60 |
| 9 | T | 32±4 | 85±13 | 97±4 |
| Neutropenia | | | | |
| 10 | S | 0 | 7±1 | 6±1 |
| 11 | T | 43±4 | 74±14 | 93±1 |
| 12 | T | 14±4 | 39±13 | 25±16 |
| Preleukaemia | | | | |
| 13 | T | 66±4 | 148±12 | 159±17 |
| 14 | T | 5±1 | 23±7 | 51±4 |
| 15 | T | 132 | 119 | 129 |
| IASA | | | | |
| 19 | T | 1 | 146 | 120 |
| AML | | | | |
| 20 | T | 0 | 17±1 | 17 |

* S = Separated, removing adherent cells. T = Unseparated total marrow.

† Each result is the no. of colonies in 4 plates (mean ± s.d.) each containing $2 \times 10^5$ nucleated marrow cells.

‡ $10^{-5}$M levamisole.

TABLE III.—*Effect of levamisole on CFU-C after liquid culture (ΔC)*

| Patient No. | Marrow cells* | Liquid cultural conditions* | CFU-C† in 20% LCM after liquid culture | ΔC‡ |
|---|---|---|---|---|
| Controls | | | | |
| 4 | T | 0 | 279±15 | 3·0 |
| | | LCM | 245±51 | 1·9 |
| | | LCM+LMS | 224±6 | 1·6 |
| 5 | T | 0 | 53±5 | 2·3 |
| | | LCM | 103±19 | 2·7 |
| | | LCM+LMS | 140±25 | 4·8 |
| 6 | S | 0 | 19±1 | 0·7 |
| | | LCM | 130±8 | 2·2 |
| | | LCM+LMS | 19±10 | 0·7 |
| 8 | S | 0 | 4±1 | 0·2 |
| | | LCM | 67±10 | 2·0 |
| | | LCM+LMS | 8±2 | 0·2 |
| 8 | T | 0 | 14±5 | 0·8 |
| | | LCM | 62±8 | 3·4 |
| | | LCM+LMS | 17±5 | 1·1 |
| Neutropenia | | | | |
| 10 | T | 0 | 0 | — |
| | | LCM | 166±52 | 11·1 |
| | | LCM+LMS | 268±14 | 10·3 |
| 11 | T | 0 | 0 | — |
| | | LCM | 160±34 | 2·2 |
| | | LCM+LMS | 202±11 | 2·2 |
| 12 | T | 0 | 0 | — |
| | | LCM | 72±9 | 1·8 |
| | | LCM+LMS | 69±16 | 2·8 |
| Preleukaemia | | | | |
| 13 | T | 0 | 70±2 | 1·1 |
| | | LCM | 168±5 | 1·2 |
| | | LCM+LMS | 172±5 | 1·2 |

* See footnote to Table II.

† $2 \times 10^5$ nucleated marrow cells tested after 7-day culture in liquid medium. (Mean ± s.d. for 2–4 replicates.)

‡ The ratio of CFU-C after liquid culture to CFU-C in 20% LCM before liquid culture (Table II).

levamisole was added in culture in the presence of LCM. There was no significant increase in CFU-C when levamisole was added to cultures without LCM. Marrow cells from patients with the "preleukaemic syndrome" yielded a similar 20% increase in CFU-C in the presence of levamisole and LCM. In contrast, no increase in CFU-C number was induced by levamisole when marrow cells from patients with acute leukaemia, neutropenia, or sideroblastosis were tested.

Marrow cells were then cultured in liquid culture with or without levamisole for 7 days, and then assayed with the usual CFU-C technique. Marrow from all patient classes tested yielded no significant increase in CFU-C after liquid culture in the presence of levamisole.

In summary, levamisole in cell culture yields increased CFU-C by modest proportions, for both normal marrow cells and those from preleukaemic patients. There is no predictable effect on CFU-C after liquid culture.

*Alteration in HMW-CSA*

Peripheral blood cells (PBL) from 2 patients with leukaemia and 3 with preleukaemia were cultured to produce LCM in the presence or absence of levamisole. The analysis of the components of HMW-CSA demonstrated one type only in all patients when LCM was produced in the usual fashion. However, when levamisole was added in culture, the cells from all

TABLE IV.—*Effect of levamisole on HMW-CSA* release*

| Patient No. | HMW-CSA moieties in medium: No LMS† | LMS in culture | LMS *in vivo*‡ |
|---|---|---|---|
| 20 (AML) | 1 | 3 | 3 |
| 21 (CML) | 1 | 3 | ND |
| 13 (Preleukaemia) | 1 | 3 | ND |
| 14 ,, | 1 | 3 | ND |
| 15 ,, | 1 | 3 | ND |

* HWM-CSA = High-mol.-wt colony-stimulating activity.

† LMS = Levamisole.

‡ One patient only received LMS orally.

patients produced 3 components of HMW-CSA. Thus, levamisole causes the cultured cells of leukaemic and preleukaemic patients to release into conditioned medium HMW-CSA similar in type to that released by normal blood cells (Table IV).

One patient with acute leukaemia was then given oral levamisole and after 5 days his peripheral blood cells produced 3 moieties of HMW-CSA, whereas before oral levamisole it produced only one component of HMW-CSA. There was no measurable clinical or haematological improvement in this patient (Table IV).

Marrow cells from 4 "control" patients were then tested for CFU-C using LCM prepared from the PBL of a single preleukaemic patient; the tests were done with conditioned medium prepared in the normal fashion, or in the presence of levamisole and dialysed. An average increase of 40% in CFU-C was noted when control marrow cells were stimulated by LCM prepared in the presence of levamisole, compared with LCM prepared in the usual way. If cells from a normal individual were used to prepare LCM, there was no comparable increase (Table V).

In summary, levamisole in culture permits PBL of leukaemic and preleukaemic patients to form LCM containing the usual 3 moieties of HMW-CSA. When a leukaemic patient was given oral levamisole, his peripheral produced LCM similar to that produced by normal PBL (*i.e.* with 3 HMW-CSA components). Finally, levamisole potentiates the release of HMW-

TABLE V.—*Effect of HMW-CSA types on CFU-C*

| Patient No. | CFU-C*: PL-1 | PL-3 | NL | NL-LMS |
|---|---|---|---|---|
| 1 | 49 | 77 | 53 | 49 |
| 6 | 75 | 92 | — | — |
| 8 | 13 | 22 | — | — |
| 9 | 18 | 26 | 30 | 28 |

* CFU-C per $2 \times 10^5$ nucleated marrow cells assayed; average of 2 plates. LCM was prepared from PBL of Preleukaemia Patient 16. LCM prepared without levamisole contained one moiety of HMW-CSA (PL-1); LCM prepared with levamisole $10^{-5}$M contained 3 components of HMW-CSA (PL-3).
NL and NL-LMS are LCM prepared from PBL of a normal donor in the absence or presence of $10^{-5}$M levamisole respectively.

CSA components into (conditioned) culture medium.

### DISCUSSION

The mechanism of action of levamisole on CFU-C is not clear. Levamisole does not appear to act directly upon granulocyte colony-forming cells, since its maximal effect generally appears to be exerted in the presence of both adherent cells and leucocyte-conditioned medium (LCM). These findings are in keeping with the study of Mahmood & Robinson (1977) which suggested that one effect of levamisole was to enhance CSA production rather than to influence CFU-C cells directly. Further understanding of the levamisole effect is provided by our data, which indicate that the drug influences the expression of high-mol.-wt components of CSA in leucocyte-conditioned medium. Contrasting the effects of LCM produced by the cells of a single preleukaemic patient and containing either 1 or 3 components of HMW-CSA, it is evident that the LCM containing 3 components is considerably more active in promoting CFU-C. Our findings suggest that CSA levamisole acts by modulation of CSA, rather than by acting directly on granulocyte colony-forming cells.

We have previously reported (Price *et al.*, 1975) that PBL from normal individuals or patients with leukaemia or preleukaemia have 3 HMW-CSA components on their cell membranes. However, LCM produced from normal cells contains 3 high-mol.-wt components, whereas we detected only one HMW-CSA component in LCM produced by cells obtained from leukaemic and some preleukaemic patients. This abnormality in HMW-CSA expression may be related to abnormal release of HMW-CSA from cell membranes, or to limitations in the detection of these components. The finding that levamisole causes cells of leukaemic and preleukaemic patients to express 3 HMW-CSA components in LCM suggests that the drug acts at the cell-membrane level, promoting the release of CSA. The observation that administration of levamisole to a leukaemic patient produces a similar effect on the ability of the patient's cells to release CSA suggests that the drug effect is not a cell-culture artefact. In the single patient tested, there was no increase in peripheral granulocyte count after 10 days of drug administration (levamisole was then discontinued because the drug was poorly tolerated by the patient). We have studied the release of HMW-CSA in leukaemic patients treated by methotrexate, busulphan or vincristine and prednisone, and have seen that only the usual single moiety of HMW-CSA was released by their cells into LCM. This observation suggests that the levamisole effect on CSA release is not common to cytotoxic drugs. In contrast, we have noted that 3 HMW-CSA components are released by the cells of leukaemic patients when remission is achieved (Price *et al.*, 1975). Thus, levamisole alters the CSA release by cells of leukaemic and some preleukaemic patients, mimicking the change when patients with leukaemia reach remission.

Brincker *et al.* (1976) have noted favourable effects from the use of levamisole in adult acute leukaemia. Lods *et al.* (1976) have used levamisole to promote restoration of normal bone marrow function after chemotherapy. Our findings in cell culture suggest that some of the beneficial effect of levamisole in this instance may be related to stimulation of haemopoietic progenitor cells. In view of other known immunostimulant effects of levamisole, it may be that the stimulatory effects on marrow are modulated through a complex series of cellular interactions in the haemopoietic and lymphoid systems.

The diagnosis of preleukaemia can now be made with a high degree of certainty by standard clinical and morphological criteria (Pierre, 1974; Linman & Bagby, 1976). The disorder may be slowly progressive, but once deterioration begins, outlook is limited and response to the usual chemotherapeutic agents is poor. For this reason, the disorder has been studied intensively, and abnormalities in cell culture have been noted (Greenberg *et al.*, 1971; Senn & Pinkerton 1972; Senn *et al.*, 1976). Linman & Bagby (1976) reported a favourable response in one preleukaemic patient which was predicted in cell culture. The present paper indicates that levamisole enhances colony formation and alters a membrane defect in the cells of some preleukaemic patients. These cultural findings suggest that levamisole would be an appropriate agent for therapeutic trial in preleukaemic states, which generally respond poorly to standard treatment regimens.

The authors are grateful to Ms Karen Benzing and Ms Penny Thompson for excellent technical help, and to the numerous physicians cooperating with us. Financial support was provided by grants from the Ontario Cancer Research Foundation (236) and the Medical Research Council of Canada (MA 1420).

### REFERENCES

AMERY, W. K., SPEAFICO, F., ROJAS, A. F., DENISSEN, E. & CHIRIGOS, M. A. (1977) Adjuvant treatment with levamisole in cancer: A review of experimental and clinical data. *Cancer Treat Rev.*, **4**, 167.

BRINCKER, H., THORLING, K. & JENSEN, K.B. (1976) Prolongation of the duration of remission in acute myeloid leukaemia (AML) with levamisole (Abst.). Scand. Haematol. Soc. Meeting, Aarhus, Sweden.

CHIRIGOS, M. A., FUHRMAN, F. & PRYOR, J. (1975) Prolongation of chemotherapeutically induced remission of a syngeneic murine leukemia by L-w 3,5,6, tetrahydro-6-phenylimidazo (2, 1-b) thiazole hydrochloride. *Cancer Res.*, **35**, 927.

GREENBERG, P. L., NICHOLS, C. W. & SCHRIER, S. L. (1971) Granulopoiesis in acute leukaemia and preleukaemia. *N. Engl. J. Med.*, **284**, 1225.

ISCOVE, N. N., SENN, J. S., TILL, J. E. & McCULLOCH, E. A. (1971) Colony formation by normal and leukemic human marrow cells in culture: Effect of conditioned medium from human leucocytes. *Blood*, **37**, 1.

LAN, S., McCULLOCH, E. A. & TILL, J. E. (1978) Cytodifferentiation in the acute myeloblastic leukemias of man. *J. Natl Cancer Inst.*, **60**, 265.

LAU, L., McCULLOCH, E. A., TILL, J. E. & PRICE, G. B. (1978) The production of hemopoietic growth factors by PHA-stimulated leukocytes. *Exp. Hematol.*, **6**, 597.

LINMAN, J. W. & BAGBY, G. C. (1976) The preleukemic syndrome: Clinical and laboratory features, natural course, and management. *Blood Cells*, **2**, 11.

LODS, J. C., DUJARDIN, P. & HALPERN, G. M. (1976) Levamisole and bone marrow restoration after chemotherapy. *Lancet*, i, 548.

MAHMOOD, T. & ROBINSON, W. A. (1977) Effect of levamisole on granulopoiesis *in vitro*. *Proc. Soc. Exp. Biol. Med.*, **156**, 359.

MESSNER, H. A., TILL, J. E. & McCULLOCH, E. A. (1973) Interacting cell populations affecting granulopoietic colony formation by normal and leukemic human marrow cells. *Blood*, **42**, 701.

MESSNER, H. A., TILL, J. E. & McCULLOCH, E. A. (1974) Specificity of interacting populations affecting granulopoiesis in culture. *Blood*, **44**, 671.

PIERRE, R. V. (1974) Preleukemic states. *Semin. Haematol.*, **11**, 73.

PRICE, G. B., SENN, J. S., McCULLOCH, E. A. & TILL, J. E. (1975) The isolation and properties of granulocyte colony stimulating activities from human peripheral leukocyte conditioned medium. *Biochem. J.*, **148**, 209.

PRICE, G. B., KROGSRUD, R., STEWART, S. & SENN, J. S. (1978) Heterogeneity and biochemistry of colony stimulating activities. In *Haematopoietic Cell Differentiation*. Eds Golde, Cline, Metcalf & Fox. New York: Academic Press. p. 417.

ROJAS, A. F., MICKIEWICZ, E., FEIERSTEIN, J. N., GLAIT, H. W. & OLIVARI, A. J. (1976) Levamisole in advanced human breast cancer. *Lancet*, i, 211.

SENN, J. S. & PINKERTON, P. H. (1972) Defective colony formation by human bone marrow preceding overt leukaemia. *Br. J. Haematol.*, **23**, 277.

SENN, J. S., PINKERTON, P. H., PRICE, G. B., MAK, T. W. & McCULLOCH, E. A. (1976) Human preleukaemia: Cell culture studies in sideroblastic anaemia. *Br. J. Cancer*, **33**, 299.

SYMOENS, J. & ROSENTHAL, M. (1977) Levamisole in the modulation of the immune response: The current experimental and clinical state. *J. Reticuloendothel. Soc.*, **21**, 175.

WARD, H. W. C. (1976) Levamisole in the treatment of cancer. *Lancet*, i, 594.

WISEMAN, L. L., SENN, J. S., MILLER, R. G. & PRICE, G. B. (1976) Stem cell characterization of neutropenia: Velocity sedimentation and mass culture analysis. *Br. J. Cancer*, **34**, 46.